# Tissue material properties, whole-bone morphology and mechanical behavior in the *Fbn1*^C1041G/+^ mouse model of Marfan syndrome

**DOI:** 10.1016/j.mbplus.2024.100155

**Published:** 2024-06-15

**Authors:** Elizabeth A. Zimmermann, Taylor DeVet, Myriam Cilla, Laia Albiol, Kyle Kavaseri, Christine Andrea, Catherine Julien, Kerstin Tiedemann, Arash Panahifar, Sima A. Alidokht, Richard Chromik, Svetlana V. Komarova, Dieter P. Reinhardt, Paul Zaslansky, Bettina M. Willie

**Affiliations:** a Research Centre, Shriners Hospital for Children-Canada, Montreal, Canada; b Faculty of Dental Medicine and Oral Health Sciences, McGill University, Montreal, Canada; c Berlin Institute of Health, Charité-Universitätsmedizin Berlin, Berlin, Germany; d Department of Nuclear Medicine, Charité-Universitätsmedizin Berlin, Berlin, Germany; e BioMedical Imaging and Therapy Beamline, Canadian Light Source, Saskatoon, Canada; f Department of Medical Imaging, University of Saskatchewan, Saskatoon, Canada; g Department of Mechanical Engineering, Memorial University of Newfoundland, St. John’s, Canada; h Department of Mining and Materials Engineering, McGill University, Montreal, Canada; i Department of Anatomy and Cell Biology, McGill University, Montreal, Canada; j Department for Operative, Preventive and Pediatric Dentistry, CC3 -Charité - Universitätsmedizin Berlin, Berlin, Germany; k Aragón Institute of Engineering Research (I3A), University of Zaragoza, Zaragoza, Spain; l Biomedical Research Networking Centre in Bioengineering, Biomaterials and Nanomedicine (CIBER-BBN), Zaragoza, Spain; m Department of Biomedical Engineering, Faculty of Engineering, University of Alberta, Edmonton, Canada

**Keywords:** Marfan syndrome, Bone composition, Bone stiffness, Strain gauging, Finite element modeling, Cortical porosity, Fourier transform infrared spectroscopy, Synchrotron phase contrast enhanced micro computed tomography

## Abstract

Marfan syndrome (MFS) is a connective tissue disorder caused by pathogenic mutations in FBN1. In bone, the protein fibrillin-1 is found in the extracellular matrix where it provides structural support of elastic fiber formation, stability for basement membrane, and regulates the bioavailability of growth factors. Individuals with MFS exhibit a range of skeletal complications including low bone mineral density and long bone overgrowth. However, it remains unknown if the bone phenotype is caused by alteration of fibrillin-1′s structural function or distortion of its interactions with bone cells. To assess the structural effects of the fibrillin-1 mutation, we characterized bone curvature, microarchitecture, composition, porosity, and mechanical behavior in the *Fbn1*^C1041G/+^ mouse model of MFS. Tibiae of 10, 26, and 52-week-old female *Fbn1*^C1041G/+^ and littermate control (LC) mice were analyzed. Mechanical behavior was assessed via in vivo strain gauging, finite element analysis, *ex vivo* three-point bending, and nanoindentation. Tibial bone morphology and curvature were assessed with micro computed tomography (μCT). Bone composition was measured with Fourier transform infrared (FTIR) imaging. Vascular and osteocyte lacunar porosity were assessed by synchrotron computed tomography. *Fbn1*^C1041G/+^ mice exhibited long bone overgrowth and osteopenia consistent with the MFS phenotype. Trabecular thickness was lower in *Fbn1*^C1041G/+^ mice but cortical bone microarchitecture was similar in *Fbn1*^C1041G/+^ and LC mice. Whole bone curvature was straighter below the tibio-fibular junction in the medial–lateral direction and more curved above in LC compared to *Fbn1*^C1041G/+^ mice. The bone matrix crystallinity was 4 % lower in *Fbn1*^C1041G/+^ mice compared to LC, implying that mineral platelets in LCs have greater crystal size and perfection than *Fbn1*^C1041G/+^ mice. Structural and mechanical properties were similar between genotypes. Cortical diaphyseal lacunar porosity was lower in *Fbn1*^C1041G/+^ mice compared to LC; this was a result of the average volume of an individual osteocyte lacunae being smaller. These data provide valuable insights into the bone phenotype and its contribution to fracture risk in this commonly used mouse model of MFS.

## Highlights

• We assessed structural effects of fibrillin-1 mutation in *Fbn1*^C1041G/+^ mouse model.
• Trabecular thickness was lower in *Fbn1*^C1041G/+^ mice than in littermate controls.
• Whole bone curvature were different in *Fbn1*^C1041G/+^ compared to littermates.
• Bone matrix crystallinity was 4 % lower in *Fbn1*^C1041G/+^ compared to littermates.
• Lacunar porosity and individual lacunar volume was lower in *Fbn1*^C1041G/+^ mice.
• We provide insights into bone phenotype and its contribution to fracture risk.

## Introduction

Marfan syndrome (MFS) results from pathogenic mutations in the fibrillin-1 gene. Fibrillinopathies (such as MFS, autosomal dominant Weill-Marchesani syndrome, geleophysic and acromicric dysplasia) cause a range of connective tissue disorders characterized by variable clinical presentations in the skeletal, cardiovascular, and ocular systems [1]. Skeletal manifestations of MFS include long bone overgrowth, arachnodactyly, and loose joints. Children and adolescents with MFS have greater bone fracture rates [2] and adults exhibit largely unexplained low bone mass [3], [4], [5], [6], [7].

The skeletal manifestations of MFS suggest that fibrillin-1 plays a major role in regulating skeletal homeostasis. Akin to other low bone mass disorders, osteopenia in MFS is likely caused by an imbalance in coordinated bone remodeling, which depends on the function of osteoblasts, osteoclasts, and osteocytes. The origin of the bone remodeling imbalance in MFS is not fully understood [8], [9], [10], [11]. Fibrillins regulate the bioavailability of growth factors of the TGF-β superfamily including TGF-β and bone morphogenetic proteins (BMPs) [12], [13], [14], [15], [16], as well as sequester osteoclastogenic RANKL [17]. TGF-β, BMPs and RANKL in turn regulate bone growth and remodeling [18], [19], [20], [21], [22], [23], [24], [25], [26], [27] specifically influencing the differentiation and functional activity of osteoblasts and osteoclasts [27], [28], [29], [30], [31], [32], [33], [34]. In addition, fragments generated during formation and degradation of fibrillin-1 were shown to demonstrate direct cell-modulatory activities [17], [35].

On the other hand, osteoporosis in MFS could be a manifestation of reduced mechanosensing by osteocytes. Osteocytes reside in micron-sized pores (lacunae) in mineralized bone forming a network of interconnected channels (canaliculi) that allow these cells to sense mechanical strain and orchestrate bone remodeling, adaptation and self-repair through regulation of osteoblasts and osteoclasts [36]. Biomechanical loads (acting on the whole bone level and measured on the bone surface with strain gauging) cause a pressure gradient that drives fluid flow through the lacuno-canalicular network of pores [37]. Fluid flow through the lacuno-canalicular network amplifies strain on the osteocytes through a number of different mechanosensory mechanisms [38]. Impaired osteocyte function and skeletal mechanoresponsiveness can lead to reduced bone mass. It is important to understand whether MFS alters the skeleton’s ability to form new bone in response to mechanical loading.

One of the most studied animal models of MFS is the *Fbn1*^C1041G/+^ mouse model, which represents a mild form of MFS severity [39]. The *Fbn1*^C1041G/+^ mouse model mimics a human heterozygous MFS missense mutation in *Fbn1*. Haploininsufficiency in heterozygous mice (i.e., lower amount of non-mutant fibrillin-1 protein) results in a Marfan phenotype, including bone overgrowth, kyphosis of the spine, and deterioration of the aortic wall [39]. *Fbn1*^C1041G/+^ mice show reduced bone mass [40]; however, the morphological and material level phenotype leading to skeletal fragility has not been examined. Female mice are investigated because fighting between male mice causes heterogeneity in background physical activity levels that influence osteocyte mechanosensation [41]. Here, we investigate the multi-scale bone phenotype of female *Fbn1*^C1041G/+^ mouse model at 10, 26, and 52 weeks of age in comparison to littermate controls (LC) in terms of osteocyte lacunar morphology, tissue material properties, whole-bone morphology, and mechanical behavior to provide insights into skeletal fragility in MFS.

## Results

### Fbn1 haploinsufficency altered whole bone morphology and curvature

**Fig. 1:**
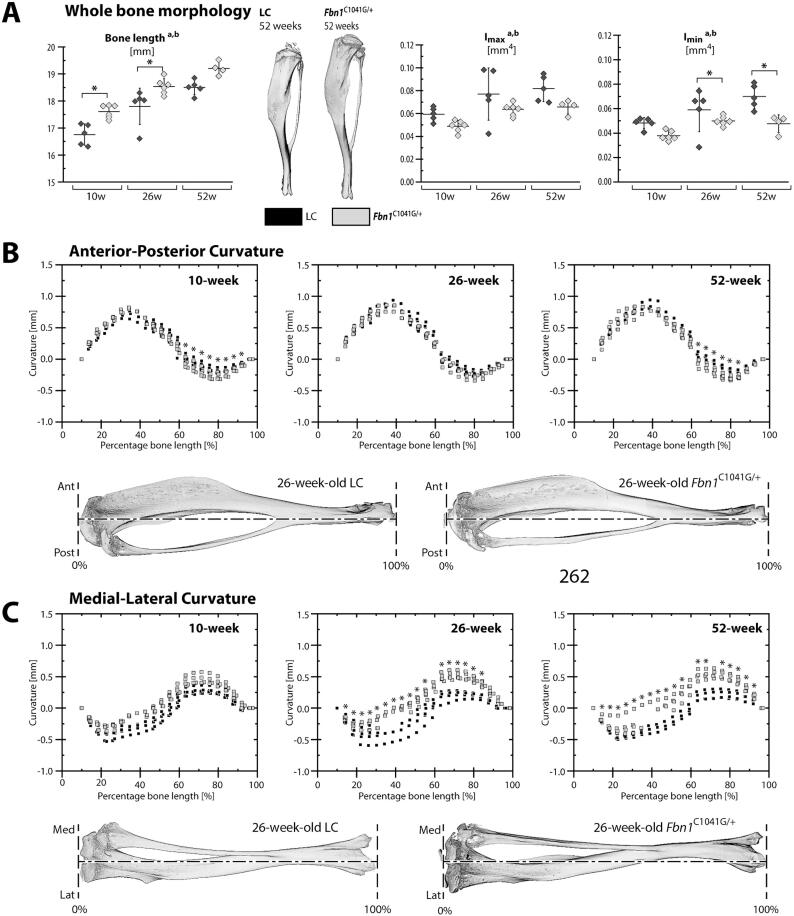
Longer bone length, smaller moment of inertia and abnormal curvature in *Fbn1^C1041G/+^* mice. Strain-gauged tibiae from female *Fbn1^C1041G/+^* and littermate control (LC) mice were imaged with μCT to measure (A) whole bone morphology and (B, C) curvature. Data for left limbs are shown as mean ± standard deviation and data for both limbs are given in Supplemental Table 2. Whole bone morphology is given in terms of bone length as well as maximum moment of inertia (I_max_) and minimum moment of inertia (I_min_) at the mid-diaphysis. Curvature along the bone length is measured in the anterior-posterior (C_ap_) and the medial–lateral (C_ml_) directions by comparing the centroid of the cross-section to the tibial axis. ANOVA main effects: ^a^genotype, ^b^age, ^c^region (percent tibial length) and interactions: ^d^genotype + age, ^e^genotype + region, ^f^region + age (Note: region only pertains to curvature analysis). Tukey-Kramer post-hoc test: *genotype. Significance for all tests was set at p ≤ 0.05.

Weight, body length, and grip strength are shown in Supplementary Table 1 for the 10, 26, and 52 week-old *Fbn1^C1041G/+^* and LC mice. Whole bone morphology and curvature were investigated in tibiae using micro computed tomography (µCT). Bone length as well as the maximum and minimum moments of inertia (I_max_ and I_min_) at the mid-diaphysis exhibited significant effects of age and genotype (Fig. 1**A,** Supplemental Table 2). The *Fbn1^C1041G/+^* mice had 3.8–4.8 % longer bones with 17–19.5 % lower I_max_ and 15–31 % lower I_min_ than LC, depending on the age group.

Anterior-posterior (AP) and medial–lateral (ML) curvature showed significant effects of age, genotype, and interactions along the bone length (Fig. 1**B,C**). The AP curvature was similar between genotype above the tibio-fibular junction, but signifcantly different in 10w and 52w old mice below the tibio-fibular junction. In the ML direction, LC mice exhibited a significantly straighter bone below the tibio-fibular junction and a more curved segment above the tibio-fibular junction than in *Fbn1^C1041G/+^* mice (Fig. 1**C**). For 10-week-old tibiae, the largest AP convexity occurred at 22 % of the tibial length from the proximal end (10w LC: C_AP_ = 0.43 ± 0.09 mm; 10w *Fbn1^C1041G/+^*: C_AP_ = 0.30 ± 0.04 mm); while in the 26 and 52-week-old tibiae, this location shifted to around 34–39 % of tibial length (26w LC: C_AP_ = 0.89 ± 0.06 mm; 26w *Fbn1^C1041G/+^*: C_AP_ = 0.80 ± 0.07 mm). In LC tibiae, the largest ML convexity (26w LC: C_ML_ = -0.48 ± 0.11 mm) appeared at 30–34 % of the tibial length from the proximal end in 26-week-old LC mice, while this location was shifted (26w *Fbn1^C1041G/+^*: C_ML_ = -0.29 ± 0.06 mm) to 22 % of the tibial length in 26-week-old tibiae.

### Fbn1 deficiency altered cortical bone structure

**Fig. 2:**
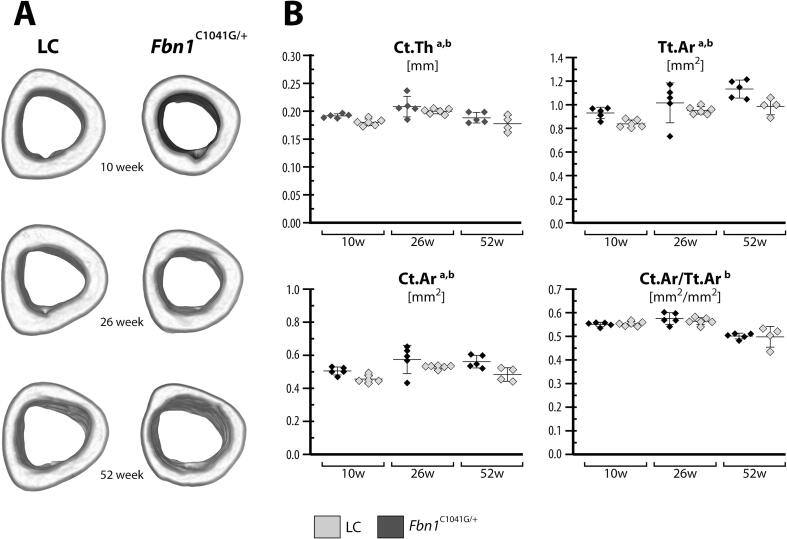
Similar cortical tibial bone microarchitecture in LC and *Fbn1^C1041G/+^* mice. (A) Representative μCT images of the left mid-diaphyseal tibiae from 10, 26, and 52 week-old female *Fbn1^C1041G/+^* and littermate control (LC) mice are shown. (B) Cortical bone microarchitecture at the mid-diaphysis was measured in terms of cortical thickness (Ct.Th), cortical area (Ct.Ar), total area (Tt.Ar) and Ct.Ar/Tt.Ar. Data are presented as mean ± standard deviation for left tibiae (data for both limbs are shown in Supplemental Table 2). ANOVA: between subject effects of ^a^genotype, ^b^age, and ^c^genotype + age. Tukey-Kramer post-hoc test: *genotype. Significance for all tests was set at p ≤ 0.05.

In the mid-diaphyseal cortical bone, ANOVA indicated significant effects due to age and genotype for cortical thickness, cortical area, and total area (Fig. 2**,** Supplemental Table 2). At the mid-diaphysis, the *Fbn1^C1041G/+^* mice have 4–6 % thinner cortices than LC mice. Also, the *Fbn1^C1041G/+^* mice had 7.8–14.0 % smaller cortical area and 6–13 % smaller total area than LC. However, these differences between genotype within female 10, 26 and 52 week-old mice were not significant according to post-hoc tests, with the exception of the cortical area fraction in the right limb of 10-week-old mice (Supplemental Table 2). We also investigated whether there was handedness in these mice. Supplemental Table 2 provides the diaphyseal microarchitectural parameters for left and right limbs for all age groups and genotypes. ANOVA was used to test for handedness; however, there was not a strong effect.

### Tissue-level strains are greater in tibiae from Fbn1 deficient mice compared to LC

**Fig. 3:**
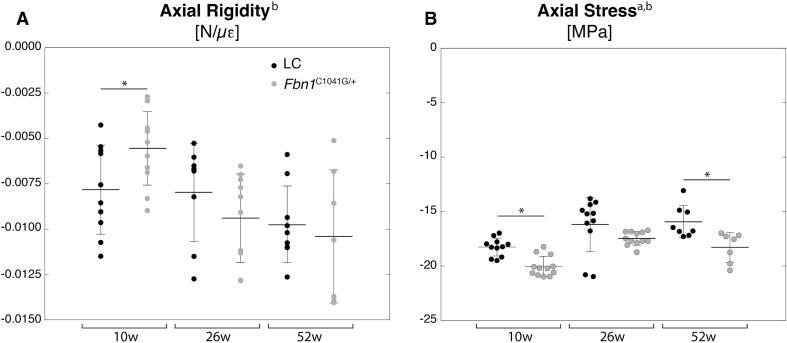
10w and 52w Fbn1^C1041G/+^ exhibit lower axial stress than LC. (A) Axial rigidity (also referred to as in vivo stiffness [42]) and (B) axial stress measured through strain gauging at the medial mid-diaphysis of 10, 26 and 52-week-old female Fbn1^C1041G/+^ and littermate control (LC) mice. Data are presented as mean ± standard deviation. ANOVA: between subject effects of ^a^genotype, ^b^age, and ^c^genotype + age. Tukey-Kramer post-hoc test: *genotype.

We examined the relationship between the applied axial compressive load and experimentally measured bone tissue deformation engendered at the tibia during in vivo tibial loading. The in vivo stiffness (also known as axial rigidity) and the axial stress are shown in Fig. 3. The axial rigidity was significantly lower in female 10w *Fbn1^C1041G/+^* mice compared to LC. Axial stress was significantly lower (implying greater compressive stress) in 10 and 52 week-old female *Fbn1^C1041G/+^* compared to their LC. The greater axial compressive stress in 10w *Fbn1^C1041G/+^* mice is reflective of their trends toward lower Ct.Ar and Ct.Th in comparison to LC.

Next finite element models (FEMs) were generated of tibiae from 26-week-old mice to estimate the local mechanical strains induced along the length of the tibia during in vivo loading. The average Young’s modulus, which was calculated for each tetrahedral element based on the attenuation coefficient of the μCT images, was 9.8 GPa for the 26-week-old female LC and 10.2 GPa for the *Fbn1^C1041G/+^.* The strain values at the strain gauge site predicted by the FEMs were similar to those measured experimentally (∼1200 µε). For the 26-week-old female *Fbn1^C1041G/+^* and LC mice, the models predicted 1202 µε and 1081 µε, respectively.

**Fig. 4:**
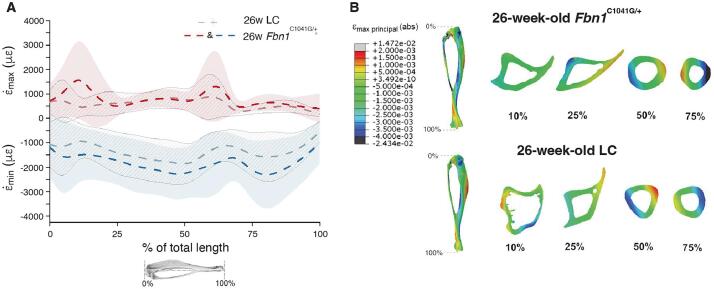
Finite element models reveal higher strains in *Fbn1^C1^*^041^*^G/+^* during in vivo tibial loading. (A) Tensile and compressive strains along the length of the tibia for the heterogeneous FEMs of the 26-week-old female *Fbn1^C1^*^041^*^G/+^* and littermate control (LC) mice. Lines represent ${\overset{¯}{\varepsilon }}_{\text{max}}$ and ${\overset{¯}{\varepsilon }}_{\text{min}}$. Shadowed regions correspond to ±SD(ε_max_) and ± SD(ε_min_). SD stands for standard deviation, which reflects the range of ε_max_ for tension and ε_min_ for compression within the cross-section. (B) Strain distribution in representative cross-sections along the bone.

The models show that the tensile average strains in the 26-week-old *Fbn1^C1041G/+^* under a load of −11.3 N are very similar to the tensile strains in the LC mice under −9.6 N load except for the metaphysis and the region located around 60 % of the total tibial length, which corresponds to the confluence of the tibia and the fibula (Fig. 4**A**). Contrarily, these load levels led to systematically higher compressive strains in the *Fbn1^C1041G/+^* than in the LC model (38 % higher in average).

### Fbn1 deficiency compromises whole-bone, but not tissue level mechanical properties

Three-point bending of tibiae from 26-week-old mice showed that *Fbn1^C1041G/+^* tibiae were weaker, although there was no significant difference in stiffness (Table 1). The maximum load of *Fbn1^C1041G/+^* tibiae was 9.5 % less than LC. This implies that there may be differences in bone material properties that contribute to bone fragility. However, because there were size and morphological differences in these bones (e.g., I_max_ and I_min_), this effect may go away.

**Table 1:** Maximum load significantly lower in *Fbn1^C1041G/+^* mice but tissue level mechanical properties not affected: Ex vivo three point bending and nanoindentation results are given for female *Fbn1^C1041G/+^* and littermate control (LC) mice. Data are presented as mean ± standard deviation. Comparison between genotype was performed using an independent T-test with significance set at *p < 0.05. Adjusted – Indicates that the parameter was adjusted for body weight. PYD – post yield displacement.

3-point-bending tests (n = 8/group)			LC			*Fbn1*^C1041G/+^	
Stiffness	N/mm	35.1	±	4.0	38.6	±	4.7
Stiffness − adjusted	N/mm	35.6	±	3.8	38.2	±	4.8
Maxium load *	N	10.3	±	0.8	9.4	±	0.9
Maxium load − adjusted *	N	10.3	±	0.8	9.4	±	0.9
PYD	mm	0.84	±	0.59	0.59	±	0.19
PYD − adjusted	mm	0.80	±	0.57	0.63	±	0.16
**Nanoindentation** (n = 7/group)			**LC**			***Fbn1*^C1041G/+^**	
Modulus	GPa	28.3	±	6.4	29.8	±	2.9
Hardness	GPa	1.1	±	0.2	1.0	±	0.1

We examined tissue level mechanical properties using nanoindentation. There were no significant difference in hardness or modulus measured between the *Fbn1^C1041G/+^* and LC mice (Table 1).

### Fbn1 deficiency alters cortical bone composition

**Fig. 5:**
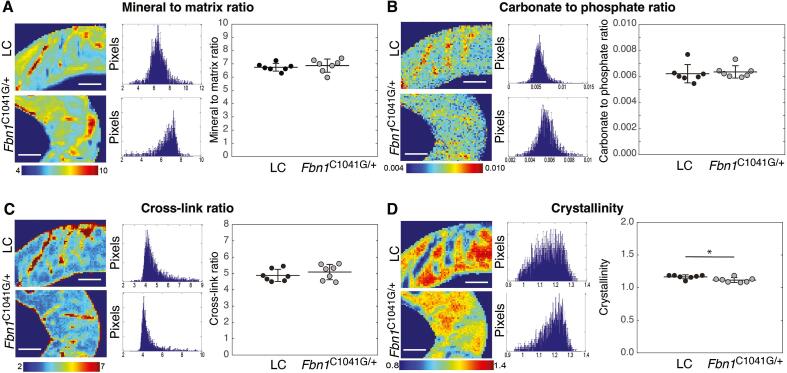
Altered bone matrix composition in *Fbn1^C1041G/+^* mice. Fourier transform infrared spectroscopy was used to measure bone composition in 26w female littermate control (LC) and *Fbn1^C1041G/+^* mice through A) mineral to matrix ratio, B) carbonate to phosphate ratio, C) cross-link ratio and D) crystallinity. Data for cortical bone at the mid-diaphysis are shown here. Data are presented as mean ± standard deviation. For each parameter, the map of the values and the distribution are shown across the entire cross-section for a representative scan. Comparison between genotype was performed using an independent T-test with significance set at *p < 0.05. Scale bar is 100 µm.

Characteristics of the bone matrix in 26-week-old mice were investigated with Fourier transform infrared spectroscopy. Representative maps and histograms of the mineral-to-matrix ratio, carbonate-to-phosphate ratio, cross-link ratio and crystallinity for both *Fbn1^C1041G/+^* and LC mice are shown in Fig. 5 and Supplemental Table 3. Bone matrix characteristics were similar in both genotypes. However, crystallinity was significantly lower in the *Fbn1^C1041G/+^* cortical bone compared to LC, which implies that mineral platelets in *Fbn1^C1041G/+^* mice have smaller crystal size and perfection than those of LCs.

### Fbn1 deficiency alters trabecular bone microstructure

**Fig. 6:**
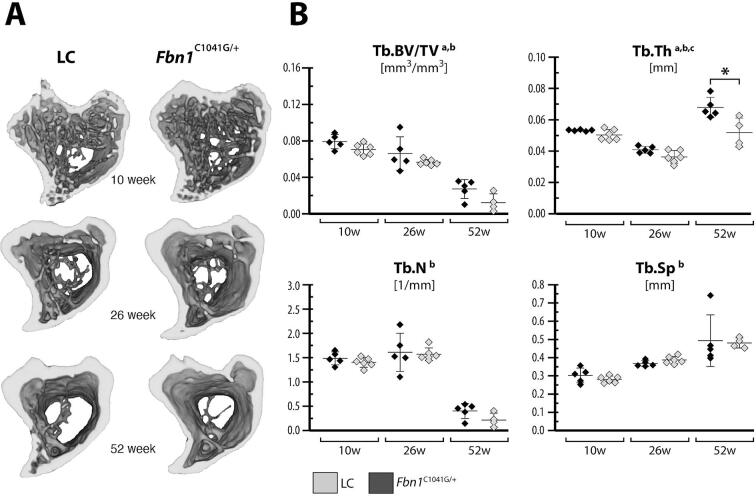
Altered trabecular bone microarchitecture in *Fbn1^C1041G/+^* mice. (A) The proximal left tibial metaphysis from female *Fbn1^C1041G/+^* and littermate control (LC) mice was imaged with μCT and the trabecular microarchitecture was analyzed at the metaphysis. Data are presented as mean ± standard deviation for left tibiae (data for both limbs are shown in Supplemental Table 2). (B) Metaphyseal trabecular microarchitecture was measured in terms of trabecular bone volume fraction (Tb.BV/TV), trabecular thickness (Tb.Th), trabecular number (Tb.N) and trabecular separation (Tb.Sp). ANOVA: between subject effects of ^a^genotype, ^b^age, and ^c^genotype + age. Tukey-Kramer post-hoc test: *genotype. Significance for all tests was set at p ≤ 0.05.

Trabecular bone microarchitecture was investigated in the tibial metaphysis. ANOVA indicated that trabecular bone volume fraction and trabecular thickness (Fig. 6) showed significant effects due to age and genotype, while the trabecular number and separation only exhibited significant differences due to age. The *Fbn1^C1041G/+^* mice had 11–55 % lower trabecular bone volume fraction and 5.5–23.6 % lower trabecular thickness although these were not significant according to post-hoc tests between genotype within age. However, we did observe significantly greater differences between genotypes in 52 week-old mice. As expected, we observed increased trabecular separation in both genotypes with increasing age. Interestingly, the trabecular number was similar between the 10 and 26-week-old female mice and only decreased dramatically in the 52-week-old female mice.

We also investigated whether there was handedness in these mice. Supplemental Table 2provides the metaphyseal microarchitectural parameters for left and right limbs for all age groups and genotypes. ANOVA was used to test for handedness; however, there was not a strong effect.

### Fbn1 deficiency results in smaller lacunae with a lower number density

**Fig. 7:**
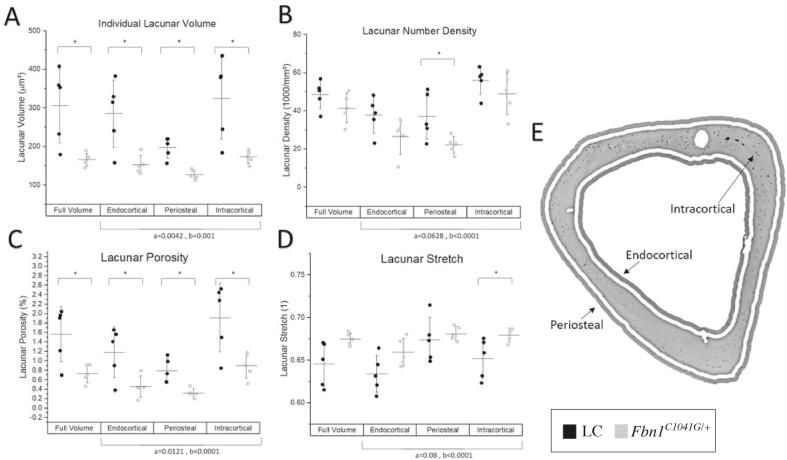
Lower osteocyte lacunar volume and bone porosity with fibrillin-1 deficiency. In 26-week-old female *Fbn1^C1041G/+^* and littermate control (LC) mice, the lacunar morphology within the right tibial diaphysis was characterized by the A) individual lacunar volume, B) lacunar number density, C) lacunar porosity, and D) lacunar stretch (i.e., the ratio between the largest and smallest eigenvector defining lacunar shape). E) The analysis was performed on four different volumes: the full volume as well as the periosteal, endocortical and intracortical regions. Data are presented as mean ± standard deviation. Significance for all tests was set at *p ≤ 0.05 for t tests, and anova. Anova results for ^a^genotype, ^b^region and ^c^interactions are shown.

Osteocyte bone cells reside within the bone matrix in interconnected pores called lacunae. Synchrotron µCT was used to investigate lacunar and vascular morphology (Fig. 7**,** Supplemental Table 4–5). The *Fbn1^C1041G/+^* mice had 85 % smaller individual lacunar volume (Fig. 7**A**) and 66 % smaller surface area in the *Fbn1^C1041G/+^* mice compared to the LC mice, with a trending lower lacunar number density (p = 0.06) (Fig. 7**B**). These differences in volume and number density translate into 114 % lower cortical lacunar porosity (Fig. 7**C**) compared to LCs in the tibial diaphysis. No difference was seen in lacunar oblateness, sphericity, or angle. Lacunar stretch (p = 0.06) was trending towards 4 % greater in *Fbn1^C1041G/+^* mice and lacunar equancy (p = 0.08) was trending towards 18 % lower in *Fbn1^C1041G/+^* mice indicating there may be small differences in the shape of the lacunae between groups. Vascular channels were 26 % closer together in the *Fbn1^C1041G/+^* mice than their LC (Supplemental Table 5), with vessel number trending towards 25 % lower in *Fbn1^C1041G/+^* mice (p = 0.07). Vascular thickness, volume, surface area and porosity were unchanged between genotypes.

When analyzing the bone based on tissue age, newer bone (periosteal and endocortical) compared to more mature intracortical bone (Fig. 7**E**), similar trends emerge. Lacunar density trended (p = 0.06) towards a lower lacunar number density in *Fbn1^C1041G/+^* compared to LC mice. In addition, there were significant differences between genotypes, with 55–88 % lower lacunar volume and a 52–72 % lower lacunar surface area, resulting in a 112–160 % lower lacunar porosity in *Fbn1^C1041G/+^* compared to LC mice. Overall porosity was lower in the periosteal and intracortical regions, with no significant difference in the endocortical region. Lacunar stretch (Fig. 7**D**) was 4 % higher in the intracortical region and lacunar equancy was 17 % lower in the *Fbn1^C1041G/+^* mice, with no significant difference in the newly formed regions.

In contrast to the diaphyseal region, we did not observe differences in lacunar, vascular, or porosity parameters between genotypes in the proximal metaphyseal tibial bone. In the metaphysis, we did measure difference in porosity parameters between regions based on tissue age (Supplemental Tables 3–4).

## Discussion

It was reported that the fracture incidence rate for a Danish cohort of 406 (196 women) people with MFS was 27.5 per 1000 person-years compared to 20.3 per 1000 person-years in the reference population [43]. Since surgical and pharmaceutical treatments to manage fatal cardiovascular events have improved [44], bone fracture related issues in people with MFS will likely become more pronounced in coming years, especially considering aging individuals with MFS. Therefore, more focus on bone in animal models of MFS is warranted to identify the source of this increased fracture risk. Despite an association of more than 3,000 mutations in FBN1 with MFS, correlations between genotype and phenotype remain difficult [45], [46]. Animal models of MFS include mouse [39], [47], pig [48], and zebrafish [49]. Multiple mouse models are available with mutations in Fbn1, including a complete *Fbn1* null, a homozygous hypomorph (mgR/mgR), deletions (mgΔ/+; GT-8/+; WMΔ/+; H1Δ/+), and missense mutations (C1041G/+; W1572C/+; D1545E/+) [50]. Although homozygous C1041G mice die during the early postnatal period, the heterozygous (*Fbn1^C1041G^*^/+^) mouse model typically live past 12 months [39]. The *Fbn1^C1041G/+^* mouse model has been widely studied, but relatively little attention has been paid to the bone phenotype. Here, we explore the multi-scale morphological and material level phenotype leading to skeletal fragility in the *Fbn1^C1041G/+^* mouse model of MFS*.*

Our results show that female 10 and 26 week-old *Fbn1^C1041G/+^* mice had longer tibial length, but there was no difference at any age in body length, weight or grip strength compared to LC mice. Walji et al. [40] observed that male 8 and 24 week-old *Fbn1^C1041G/+^* mice had longer tibial length and body length than WT mice. Long bone overgrowth and increased height are commonly observed in individuals with MFS [51]. Mice differ from humans in that their growth plates do not cease growth; however, the rate of longitudinal bone growth slows at sexual maturity. This could be why *Fbn1^C1041G/+^* mice had longer tibial length at 10 and 26 weeks but not 52 weeks.

We analyzed porosity at the tibial diaphysis of female 26-week-old mice using synchrotron tomography. Although total porosity and osteocyte lacunar number density were not different between *Fbn1^C1041G/+^* and LC mice, we did observe significantly lower lacunar porosity accompanied by 66 % lower surface area and an 85 % smaller individual lacunar volume in *Fbn1^C1041G/+^* compared to LC mice. While osteocyte lacunar shape was not altered in newly formed bone in the periosteal and endocortical regions, there were significant differences in lacunar equancy and oblateness in the older intracortical bone. This could indicate osteocyte shape is altered in *Fbn1^C1041G/+^* mice over time, which could impact osteocyte mechanosensation [38]. The total porosity results in female mice are quite different to what has been reported in women with MFS. Folkstad et al. [51] reported individuals with MFS have significantly greater cortical porosity (Radius: 1.900 %, Tibia: 4.850 %) compared to controls (Radius: 0.015 %, Tibia: 0.049 %) in the radius and tibia, respectively. Further stratification of the data found that pre-menopausal women with MFS had 4.2 % cortical porosity compared to 0.045 % in controls. Cortical porosity measurements in humans (via high resolution peripheral quantitative computed tomography at a voxel size of 82 µm) reflect vascularity and resorption channels created by intracortical remodeling. However, mice (unlike humans) do not normally undergo intracortical remodeling, during which osteoclasts bone cells resorb a cylindrical cone within the cortex followed by osteoblast bone formation on the surface of the resorption cavity around a central vascular channel. Intracortical remodeling produces Haversian canals (200–300 µm in diameter), sometimes called secondary osteons. Here, our synchrotron-based porosity measures in mice (voxel size of 0.72 µm) reflect vascularity and osteocyte lacunar porosity. The greater cortical porosity in humans with MFS is likely contributing to increased fracture risk; these results highlight the limitations of mice as a model for a disorder with a presentation of high intracortical porosity. Additional studies are warranted to examine lacunar porosity in humans as well as porosity in male mice, at other ages and in other MFS mouse models.

Somewhat surprisingly, the cortical bone microstructure in the mice was similar between genotypes. Although we observed that *Fbn1^C1041G/+^* mice had thinner cortices and smaller cortical area and total area compared to LC mice, none of these effects reached significance in female mice, except for the cortical area fraction in the right limb of 10-week-old mice, which was significantly lower in *Fbn1^C1041G/+^* compared to LC mice. Walji et al. [40] reported in male 24-week-old mice, significantly lower cortical, total, and medullary area in *Fbn1^C1041G/+^* compared to WT mice. In terms of the trabecular microstructure, we only observed significantly thinner trabeculae in female 52-week-old *Fbn1^C1041G/+^* compared to LC mice. Walji et al. [40] reported in male 24-week-old *Fbn1^C1041G/+^* mice significantly lower trabecular bone volume fraction accompanied by fewer and thinner trabeculae. Other murine models follow similar trends as the *Fbn1^C1041G/+^* mice. Artegea-Solis et al. [52] found lower cortical area in *Fbn1*^mgR/mgR^ mice compared to LC, but in this mouse model, there was no genotypic difference in trabecular bone microarchitecture.

Folkestad et al., [51] investigated bone density and microarchitecture in adults with and without MFS using dual x-ray absorptiometry (DXA) and high resolution peripheral quantitative computed tomography (HR-pQCT). Adults with MFS were observed to have lower DXA-measured areal bone mineral density at the spine and hip in comparison to healthy controls. Furthermore, HR-pQCT measures at the distal radius and tibiae found lower total, cortical and trabecular volumetric bone mineral density in adults with MFS compared to controls. In terms of microarchitectural parameters both men and women with MFS have greater total area and trabecular area at both skeletal sites, with lower cortical area, thickness, trabecular thickness and trabecular number in comparison to healthy controls [51]. The female *Fbn1^C1041G/+^* mice do not recapitulate the striking differences in cortical and trabecular bone microstructure observed in humans with MFS.

One limitation of our study is that we did not analyze male mice. Although we cannot directly compare sex at the same age, data reported by Walji et al. [40] of 8 and 24-week-old male mice suggest that male *Fbn1^C1041G/+^* mice have a more pronounced bone phenotype in terms of cortical and trabecular microstructural deficits compared to females. Further analyses of sex differences in the bone phenotype at various hierarchical levels is warranted to determine if males have more similar phenotype to humans with MFS in terms of cortical and trabecular bone microstructure.

We also observed similar structural (i.e., strength and stiffness) and material (i.e., hardness and elastic modulus) mechanical properties between genotypes. In contrast, we observed striking differences in whole bone morphology (i.e., lower moment of inertia) and curvature between genotypes. The altered curvature (straighter below tibio-fibular junction and more curved above) was most striking in the 10 week and 52-week-old *Fbn1^C1041G/+^* mice compared to LC mice.

In 26-week-old mice, we observed similar Young’s modulus estimated by FEM for the *Fbn1^C1041G/+^ (*10.2 GPa) and the LC mice (9.8 GPa), which was in line with what we observed for nanoindentation modulus and whole bone stiffness between the genotypes. However, higher compressive loads (17 % higher) were needed in the 26w *Fbn1^C1041G/+^* mice to engender comparable strain levels (+1200με) at strain gauge site. Mechanical properties of the tibia seem not to explain the differences, since no significant differences were observed either in the material properties, cross-sectional area, or the antero-lateral curvature. However, variations in the position of the strain gauge along the tibial length may also explain the mechanical behavior in the *Fbn1^C1041G/+^* mice. The FEMs reveal that these loads led to comparable tensile strains not only at the strain gauge site but all along the tibia except for the metaphysis and the distal tibiofibular region, but the compressive strains were higher (38 % in average) in the 26-week-old *Fbn1^C1041G/+^* than in the age matched LC. This suggests that an identical load would lead to lower tensile strains in the *Fbn1^C1041G/+^* compared to the LC mouse, but the compressive strains would still be higher or at least equal. A different distribution of the load between the tibia and the fibula seems to play a role in this phenomenon, since the fibula bears a higher percentage of the external load in the 26-week-old *Fbn1^C1041G^*^/+^, leading to higher strains in the fibula and lower tensile strains in the anteromedial tibia (Fig. 4**B**).

In terms of cortical bone composition, only the mineral crystallinity was significantly different, but not mineral-to-matrix, carbonate-to-phosphonate or crosslinking ratios. Arteaga-Solis et al. [52] reported no difference in cortical bone carbonate-to-phosphonate ratio, crosslinking or crystallinity, but a trend of lower mineral-to-matrix ratio in Fbn1^mgR/mgR^ compared to WT mice. Crystallinity is a measure of apatitic to non-apatitic domains that generally increases with tissue age. Taylor et al. [53] show that crystallinity is linearly associated with crystal length. In cortical bone of TGF- β1 null mice, mineral crystallinity was reduced along with collagen maturity and mineral content [54]. Thus, consistent with our results, the deficiency in fibrillin could alter the TGF- β profile and lead to reduced mineral maturation.

## Conclusions

Although less is known about the bone phenotype in humans with MFS, the bone phenotype we observed in female *Fbn1^C1041G/+^* mice did not largely resemble that reported in women with MFS. Further studies in male *Fbn1^C1041G/+^* mice at various ages are warranted to determine if it better recapitulates the bone phenotype observed in people with MFS. Here, we investigate the multi-scale bone phenotype of the female *Fbn1*^C1041G/+^ mouse model at 10, 26, and 52 weeks of age in comparison to littermate controls (LC) to provide insights into skeletal fragility in MFS. We observe striking differences in the *Fbn1^C1041G/+^* mice in whole bone geometry, moment of inertia, and trabecular thickness; however, cortical bone microarchitecture was similar in *Fbn1*^C1041G/+^ and LC mice. At the tissue level, bone mineralization, modulus and hardness were similar; however, mineral platelets had greater crystallinity than *Fbn1*^C1041G/+^ mice. Thus, the differences in strain distribution observed with strain gauging and finite element models is likely due to differences in geometry and not tissue properties. Lacunar porosity and individual lacunar volume were smaller in *Fbn1*^C1041G/+^ than LC mice implying that osteocyte mechanosensing may be affected by fibrillin-1 deficiency.

## Experimental procedures

### Animals

Female *Fbn1^C1041G/+^* mice (Jackson Laboratory: B6.129-*Fbn1^tm1Hcd^*/J) and their littermate controls (LC) were investigated at 10, 26, and 52 weeks of age. Mice were bred and housed at the animal facility at Shriners Hospital for Children-Canada under McGill animal use protocol 2016–7821 and 2014–7561. Tibiae from a first group of 10, 26, and 52-week-old mice were used for strain gauging, finite element analysis and micro computed tomography imaging. A second group of 26-week-old mice was euthanized and tibiae were fresh frozen. One tibia was used for ex vivo mechanical testing and the other tibia was embedded in methyl methacrylate for material property and cortical porosity analyses (Fourier transform infrared spectroscopy, nanoindentation, and synchrotron imaging).

### Mouse characteristics and grip strength

Age, weight, and body length were documented. Forelimb grip strength was measured using a grip strength meter (BIO-GS3, Bioseb, Pinellas Park, FL, USA). Briefly, mice were positioned over the grip strength meter such that they instinctively gripped the wire with their two front limbs. The mice were gently pulled back by the base of their tail until they had completely separated from the grid. The grip strength meter recorded the maximum force (grams). Measurements were performed 5 times with a two-minute break in between measurements.

### In vivo strain gauging

In vivo strain gauging was performed on 10, 26, and 52-week-old female *Fbn1^C1041G/+^* and LC mice (n = 5–7 mice/age/genotype). Strain gauging of both tibiae was performed on the medial surface of the tibial midshaft to determine the relationship between applied tibial compressive loads and bone tissue deformation using established methods [55]. Mice were anesthesized with isofluorane anesthesia. Skin and muscle on the medial lateral surface of the tibia was separated to reveal the bone. A uniaxial strain gauge (EA-06-015LA-120, Micromeasurements, Raleigh, NC, USA) and wires (Phoenix Wire Inc., South Hero, VT), aligned with the bone’s long axis, was fixed to the bone surface at the tibia midshaft with a drop of cyanoacrylate glue. While the mouse was under anesthesia, its tibia was placed in a mechanical testing device (ElectroForce TestBench, TA Instruments, New Castle, DE, USA) to undergo in vivo tibial loading at various load levels [56]. A range of in vivo cyclic compressive loads (peak loads ranging from −2 to −12 N) were applied to the tibia in an ascending and then descending manner using a triangle waveform at 4 Hz, characterized by 0.15 sec of symmetric active loading/unloading with a 0.10 sec rest insertion between the load cycles [57]. The strain measurements were recorded simultaneously at 2.5 kHz and once repeatable peak strains were reached, lead wires were cut, with the gauges left intact on the bone. Mice were euthanized with isofluorane anesthesia and carbon dioxide. Tibiae were dissected, fixed in formalin for two days and stored in PBS at 4 °C. Effective axial rigidity, also referred to as in vivo tibial stiffness [42], [55], was calculated as the change in load over the change in strain during the loading portion of the waveform and averaged across four consecutive load cycles to obtain a mean axial rigidity per mouse. To understand how morphological changes with age and genotype affected in vivo tibial stiffness, axial stresses induced by a 9 N compressive axial load were calculated at the level of the strain gauge. A single load was used for all age and genotype groups to directly compare the effects of differences in bone morphology on load- induced stresses and bone stiffness. The axial stress (σ^ax^) induced by the compressive load was calculated as σ^ax^ = P/Ct.Ar, where the load, P, was set to −9N and Ct.Ar is the bone cross-sectional area at the level of gauge attachment.

### Bone density and microstructure

Strain-gauged tibiae were imaged with micro computed tomography (μCT) to analyze metaphyseal and diaphyseal bone geometry and microarchitecture. Samples were wrapped in gauze and transferred to PBS filled tubes. The tibiae were then imaged with x-ray tube set at 55 kV and 81 µA, with a voxel size of 10 µm^3^ using a 0.25-mm aluminium filter (SkyScan 1276 and SkyScan 1172, Bruker). Samples were imaged at 180 degrees at a rotation step of 0.3 degrees with a frame averaging of 3. During reconstruction, all samples were corrected for ring artifacts, post-alignment, and beam hardening using the software suite provided by the scanner. All images were aligned manually in the same position using DataViewer (Bruker). A volume of interest (VOI) was positioned at 50 % of the bone length with a height of 5 % of the bone length to capture diaphyseal cortical bone. Additionally, a metaphyseal VOI was positioned 100 μm below the growth plate to capture secondary spongiosa and metaphyseal cortical bone, with a height of 10 % of the bone length. CT Analyser software (version 1.16.4.1 Bruker) was used to quantify the bone mass, microstructure, and morphology. Standards of 250 and 750 mg hydroxapatite (HA) per cubic centimeter were scanned at the same settings as the bone samples and used to calibrate the grey values. A threshold of 800 mg HA/ cm^3^ was used as a global threshold for diaphyseal and metaphyseal cortical bone, while a threshold of 500 mg HA/cm^3^ was used as a global threshold for the metaphyseal trabecular bone. In the cortical regions, the total area (Tt.Ar, mm^2^), cortical area (Ct.Ar, mm^2^), cortical area fraction (Ct.Ar/Tt.Ar, mm^2^/mm^2^), and cortical thickness (Ct.Th, mm) were calculated from a three-dimensional analysis, while the average maximum moment of inertia (I_max_, mm^4^), and average minimum moment of inertia (I_min_, mm^4^) were calculated by averaging the two-dimensional data across the volume. In the metaphyseal trabecular region, the bone volume fraction (BV/TV, mm^3^/mm^3^), trabecular number (Tb.N, 1/mm), trabecular thickness (Tb.Th, μm) and trabecular separation (Tb.Sp, μm) were measured.

### Whole bone curvature

Whole bone curvature was measured and compared between both genotypes and all ages. The μCT images were aligned manually in the same position using DataViewer (Bruker) and the global threshold was applied. Curvature was calculated with a custom-made Matlab script, using a procedure previously described [58]. Briefly, two reference slices were identified: 1) slice at 10 % tibial length in the proximal metaphysis and 2) slice in the distal tibial metaphysis where the medial side of the tibia becomes convex. The fibula was automatically removed from the image. The tibial axis was determined by calculating the position of a straight line between the centroids of these two reference slices. The curvature was measured at 20 points along the bone length by comparing the position of the tibial axis to the centroid.

### Finite element analysis

We developed microCT-based finite element models (FEMs) to examine tissue-level strains engendered across the entire bone during in vivo tibial loading. Our FEM approach was previously validated in 10, 26, and 78 week old C57BL/6 mice [59], [55]. Whole bone geometry was acquired from 26-week-old female *Fbn1^C1041G/+^* and LC tibiae (n = 1/genotype), based on ex vivo μCT scanning of the strain-gauged whole tibiae, at an isotropic voxel resolution of 9.91 μm (Skyscan 1276, Kontich, Belgium; 100 kVp, 100 µA, 360°, 0.3° rotation step, 3 frame averaging). Our model used a compression load applied through the knee with an inclination respect to the bone axis of 10 degrees, based on our previous studies in female C57Bl/6 mice [58], [59], [55]. Our model allowed surface nodes on the distal part of the bone to move in anterior-posterior direction, but not in the medial–lateral (malleoli constrains this movement) and to rotate around the medial–lateral axis of the tibia, the axis of the articulatio talocruralis (hinge-joint). We considered bone properties to be linear elastic and isotropic, with a poissońs ratio set to 0.35 for all models. We assigned a Younǵs modulus (E) to each tetrahedral element of the heterogeneous tetrahedral FEMs; this was estimated with a power-law relationship between the stiffness E_i_=(μ_i_/μ_TMD=1.51_)^1.5^*17 GPa and linear attenuation coefficient (μ). We assigned all elements with a TMD ≥ 1.51 g*cm^−3^ a maximal E equal to 17 GPa. We previously determined the linear relationship between TMD and μ using two hydroxyapatite (HA) phantoms. Our material properties assignment and meshing methods have been previously described [58], [59]. We calculated the predicted strain value at the gauge site for each tibia. This was done by averaging the strain in the longitudinal direction of the strain gauge at its mounting position, which was visible on the scans. We calculated the predicted strains in bone tissue by the maximum absolute value between the maximal (tensile, ε_Max_) and minimal (compressive, ε_Min_) principal strains for each element within the FEMs*.*

### Whole-bone mechanical properties determined by three-point bending tests

The right tibiae were dissected from 26-week-old *Fbn1^C1041G/+^* and their LC (n = 8/genotype) mice and wrapped in saline soaked gauze and frozen until the time of testing. Tibiae were tested to failure in three-point bending using a servohydraulic testing system (Instron, Next Generation 6800 Series, MA) with a 25 lbs (111 N) load cell, an 8.5 mm span and a displacement rate of 0.1 mm/s. The load was applied to the anterior surface of the tibial midshaft. Load and displacement data were simultaneously collected at 20 Hz. The following parameters were reported: Stiffness (N/mm), Maximal (Max) Load (N), and Post Yield Displacement (mm).

### Fourier transform infrared (FTIR) imaging

FTIR imaging was performed to characterize the composition of the bone mineral and matrix in MFS. Left tibiae from 26-week-old *Fbn1^C1041G/+^* and their LC (n = 5/genotype) were fixed in formalin for two days, dehydrated in an ascending ethanol series and embedded in methyl methacrylate (MMA). In the tibial metaphysis, 1-μm thick slices of bone were sectioned using a microtome. The sections were mounted on barium fluoride infrared windows and imaged with a FTIR microscope (Perkin Elmer, Spotlight 100 imaging system, MA). Spectra were collected with a 4-cm^−1^ spectral resolution and a 6.25-μm spatial resolution on the metaphyseal trabecular bone and the posterior metaphyseal cortical bone. Spectra were processed with ISYS software (Spectral Dimensions, Olney, MD). Water vapor and MMA were first subtracted, followed by calculation of the following parameters: mineral-to-matrix ratio (area ratio of v1, v3 phosphate band from 900-1200 cm^−1^ to amide I band at 1590–1712 cm^−1^), carbonate-to-phosphate ratio (area ratio of carbonate band from 855 to 890 cm^−1^ to phosphate band), crystallinity (peak intensity ratio of subbands 1030 and 1020 cm^−1^), collagen maturity (peak intensity ratio of subbands 1660 and 1690 cm^−1^), and acid phosphate content (peak height ratio of subbands 1096 and 1128 cm^−1^) [60].

### Synchrotron x-ray μCT

We performed synchrotron-based phase contrast enhanced *μ*CT at the BioMedical and Therapy (BMIT-BM) beamline of the Canadian Light Source (Saskatoon, Canada)[61] on the left tibiae from 26-week-old *Fbn1^C1041G/+^* mice and their LC (n = 6/genotype). The isotropic effective pixel size of the images was set to 0.72 µm resulting in a field of view of 1.84x 1.56 mm. White beam from a bending magnet was filtered with 0.88 mm aluminum and 0. 8 mm molybdenum to have an average energy of 26 keV. A white-beam microscope (Optique peter, France) equipped with a 10x objective, a sCMOS camera (Edge 5.5, PCO, Germany), and 13 µm thick LSO scintillator was used for detection of X-rays. Sample to detector distance was 15 mm. Depending on the dimensions of the sample a 180 degree scan with 3000 projections or 360 degree scan (i.e., half-acquisition scan to double FOV) with 6000 projections were collected at 100 ms exposure time per projection[61].

Using Nrecon v2.2 (Bruker, Massachusetts, USA) phase contrast enhancement was done using a delta beta ratio of 300 and restoration gauss width and stabilizer of 0.1, with a sample-to-detector distance of 10 mm. Reconstructions were then performed using a ring reduction algorithm.. Image analysis was performed using Xamflow 1.8.8.0 (Lucid Concepts, Zurich, Switzerland). Images were filtered with a gaussian filter of sigma 0.6 and a support value of 2 to reduce noise in the image. This resulted in clean images that could be directly thresholded using an isodata based auto thresholding algorithm. Lacunae were segmented and detected using an algorithm that sorted them by size and shape, to exclude vascular pores [62]. The bone was then sectioned into different regions for analysis. The sections of periosteal and endosteal bone within 20 µm of the surface were isolated [63], and intracortical bone 20 µm away from these regions was also isolated. Lacunae in each of these regions were analyzed together to look at local and global trends in shape, orientation, and number density. A lacunar count was calculated by identifying pores between 50 and 2000 µm^3^ [62]. This was used to calculate lacunar number density as lacunar count normalized to bone volume. The volume and surface area of each lacuna was calculated, and these were used to determine the total porosity of the bone that was created by lacunae. To determine lacunar shape, each lacuna was fit with 3 eigenvectors identifying the 3 principal axis of the pore. The angle of the main axis relative to the main axis of the bone was used to calculate lacunar angle. Lacunar sphericity was calculated as the surface area to volume ratio. Lacunar equancy was calculated as an aspect ratio between the smallest and largest eigenvector, with lacunar stretch being a normalized version of the aspect ratio. Lacunar oblateness was used as a measure between 0 and 1 to quantify whether the lacunar shape was rod-like or plate-like.

### Nanoindentation

Measurements were conducted on left tibiae from 26-week-old *Fbn1^C1041G/+^* and their LC (n = 7/genotype) with a nanoindentation device (Hysitron Inc., Minneapolis, USA) using a Berkovich diamond indenter tip. The MMA-embedded tibiae were cut perpendicular to the long bone axis at the mid-diaphysis and polished with ascending grades of silicon carbide paper and alumina suspension to get a smooth, planar surface. The mechanical properties were evaluated over an area of approximately 30 x 100 µm per sample on the posterior-lateral surface, consisting of a grid of 10 x 3 measurement points, with a step size of 10 µm. The starting point of the measurement was specified using a light microscope. The loading of each indentation consisted of a loading phase (loading rate 1000 µN/s), a resting phase (loading 5000 µN for 60 s), and an unloading phase (unloading rate 400 µN/s). Hardness H and indentation modulus E_r_ were calculated as previously described and measurement points with indentation moduli below a threshold of 10 GPa were excluded, since they were attributed to the embedding material or next to holes within the bone.

### Statistics

For µCT and whole bone curvature, the effect of age (10, 26, 52 weeks old), genotype (*Fbn1^C1041G/+^* and LC), and limb (left or right tibia) as well as interactions between these terms was assessed using an ANOVA (SAS 9.4, Cary, USA). Analyses of µCT parameters of only left limbs were also performed examining effect of age, genotype, and interactions. Tukey-Kramer post-hoc test was used to analyze significant interaction terms. For FTIR measurements, independent T-tests were performed. Lacunar, vascular, and porosity measurements were analyzed using independent t-tests to look at the full volume, and an ANOVA with Tukey-Kramer post-hoc tests to look for significant interaction terms. Unless otherwise indicated, results reported were significant (p < 0.05) and presented as mean ± standard deviation. The percent change values were calculated as (A –B)/B *100.

## CRediT authorship contribution statement

**Elizabeth A. Zimmermann:** Writing – review & editing, Writing – original draft, Visualization, Project administration, Methodology, Investigation, Formal analysis, Data curation, Conceptualization. **Taylor DeVet:** Writing – review & editing, Visualization, Formal analysis, Data curation. **Myriam Cilla:** Writing – review & editing, Formal analysis, Data curation. **Laia Albiol:** Writing – review & editing, Formal analysis, Data curation. **Kyle Kavaseri:** Writing – review & editing, Formal analysis, Data curation. **Christine Andrea:** Writing – review & editing, Formal analysis, Data curation. **Catherine Julien:** Writing – review & editing, Project administration, Formal analysis, Data curation. **Kerstin Tiedemann:** Writing – review & editing, Formal analysis, Data curation. **Arash Panahifar:** Writing – review & editing, Formal analysis, Data curation. **Sima A. Alidokht:** Writing – review & editing, Formal analysis, Data curation. **Richard Chromik:** Supervision, Data curation. **Svetlana V. Komarova:** Writing – review & editing, Supervision, Funding acquisition. **Dieter P. Reinhardt:** Writing – review & editing, Supervision, Funding acquisition. **Paul Zaslansky:** Writing – review & editing, Formal analysis, Data curation. **Bettina M. Willie:** Writing – review & editing, Writing – original draft, Supervision, Project administration, Investigation, Funding acquisition, Formal analysis, Data curation, Conceptualization.

## Declaration of competing interest

The authors declare that they have no known competing financial interests or personal relationship that could have appeared to influence the work reported in this paper.

## Data Availability

Data will be made available on request.
