# Corrigendum to “Tissue material properties, whole-bone morphology and mechanical behavior in the *Fbn1*^C1041G/+^ mouse model of Marfan Syndrome” [Matrix Biol. Plus 23 (2024) 100155]

**DOI:** 10.1016/j.mbplus.2025.100176

**Published:** 2025-06-16

**Authors:** Elizabeth A. Zimmermann, Taylor DeVet, Myriam Cilla, Laia Albiol, Kyle Kavaseri, Christine Andrea, Catherine Julien, Kerstin Tiedemann, Arash Panahifar, Sima Alidokht, Richard Chromik, Svetlana V. Komarova, Dieter P. Reinhardt, Paul Zaslansky, Bettina M. Willie

**Affiliations:** a Research Centre, Shriners Hospital for Children-Canada, Montreal, Canada; b Faculty of Dental Medicine and Oral Health Sciences, McGill University, Montreal, Canada; c Department of Mechanical Engineering, University of Zaragoza, Zaragoza, Spain; d Julius Wolff Institute and BIH Center for Regenerative Therapies, Berlin Institute of Health and Charité - Universitätsmedizin Berlin, Berlin, Germany; e BioMedical Imaging and Therapy Beamline, Canadian Light Source, Saskatoon, Canada; f Department of Medical Imaging, University of Saskatchewan, Saskatoon, Canada; g Department of Mechanical Engineering, Memorial University of Newfoundland, St. John’s, Canada; h Department of Mining and Materials Engineering, McGill University, Montreal, Canada; i Department of Anatomy and Cell Biology, McGill University, Montreal, Canada; j Faculty of Dentistry, Berlin Institute of Health and Charité - Universitätsmedizin Berlin, Berlin, Germany

The authors previously mentioned that the “mice were bred and housed at the animal facility at Shriners Hospital for Children-Canada under McGill animal use protocol 2016–7821 and 2014–7561.” However, the authors regret not previously stating that the animal use was approved by the McGill Animal Compliance Office and the Facility Animal Care Committees at Shriners Hospital for Children-Canada (protocols 2016–7821 and 2014–7561), in accordance with the Canadian Council on Animal Care.

In addition, the legend for Figs. 2 and 6 were incorrectly labelled; the littermate controls (LC) should have been labelled as black and the Fbn1^C1041G/+^ as grey. The authors would like to apologise for any inconvenience caused.

