# Supplementary material for: Tissue material properties, whole-bone morphology and mechanical behavior in the Fbn1C1041G/+ mouse model of Marfan syndrome
Source: Matrix Biol Plus. 2024 Jun 15;23:100155. doi: 10.1016/j.mbplus.2024.100155 (PMC11267061; doi:10.1016/j.mbplus.2024.100155)

**Supplementary Table 1: Characteristics of mice.** Weight, body length, and grip strength (absolute & normalized by body weight) were measured prior to euthanasia in 10, 26, and 52 week-old female *Fbn1^C1041G/+^* and LC mice. Data are presented as mean ± standard deviation. ANOVA assessed differences between ^a^genotype, ^b^age and ^c^genotype*age. Tukey-Kramer post-hoc test: *genotype. Significance for all tests was set at p ≤ 0.05.

*
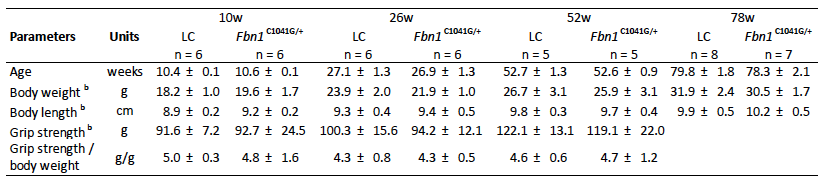
*

**Supplemental Table 2: Trabecular and cortical bone microarchitecture in the tibiae.** Bone microarchitecture was measured in diaphyseal cortical bone, metaphyseal cortical bone and metaphyseal trabecular bone in 10w, 26w and 52w female littermate control (LC) and *Fbn1^C1041G/+^* mice. Data are presented as mean ± standard deviation. ANOVA main effects: ^a^genotype (*Fbn1^C1041G/+^*, LC mice), ^b^age (10, 26, 52 week old), ^c^limb (left, right tibiae) and interactions: ^d^genotype+age, ^e^limb+genotype, ^f^limb+age. Tukey-Kramer post-hoc test: *genotype within each age and limb, §10w vs 26w, #10w vs 52w, † 26w vs 52w. Significant differences between right and left limbs determined by a paired t-test is indicated by $. Significance for all tests was set at p ≤ 0.05.


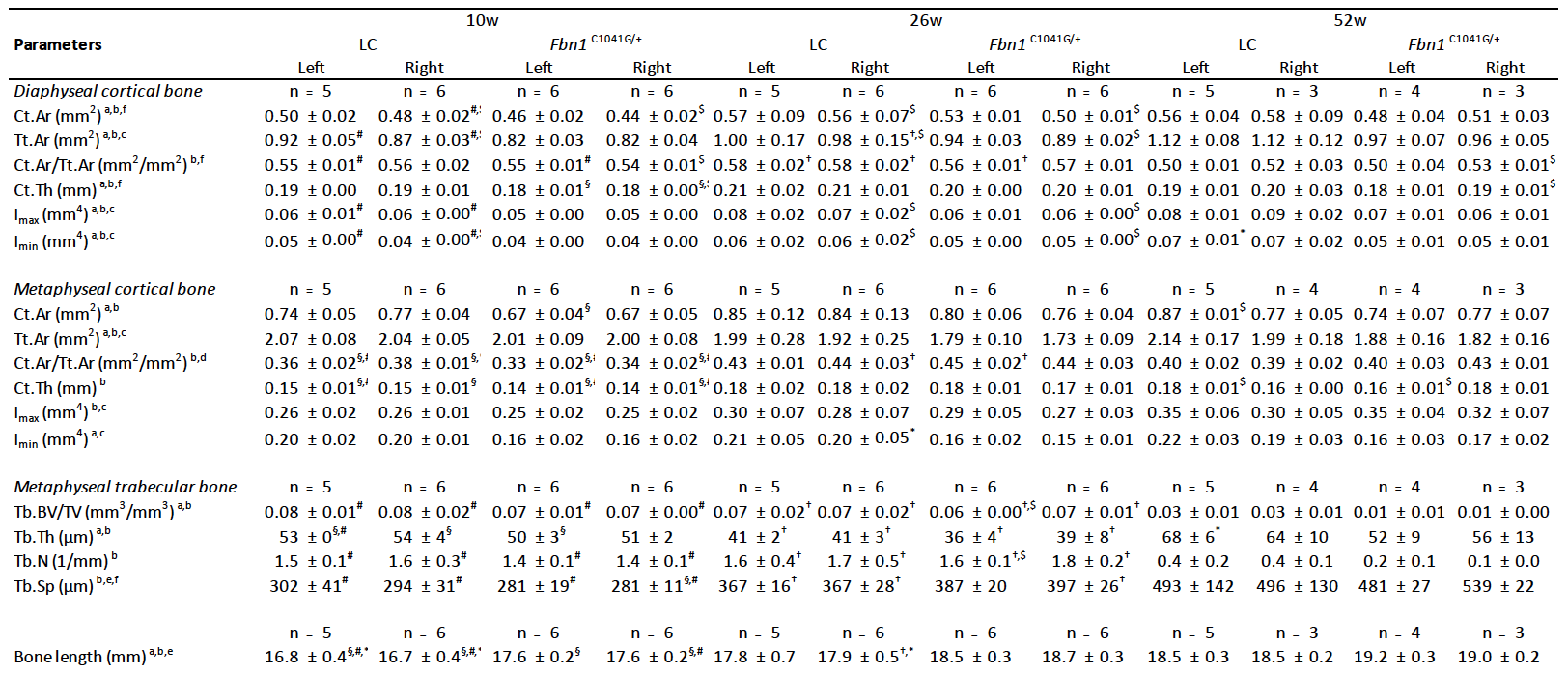


**Supplemental Table 3. FTIR parameters:** Fourier transform infrared spectroscopy (FTIR) data are presented as mean ± standard deviation from female 26-week-old *Fbn1^C1041G/+^* and littermate control (LC) mice. Comparison between genotype was performed using an independent T-test with significance set at *p < 0.05. MMR: mineral-to-matrix ratio. CPR: carbonate-to-phosphate ratio. APC: Acid phosphate content. Het: Heterogeneity.


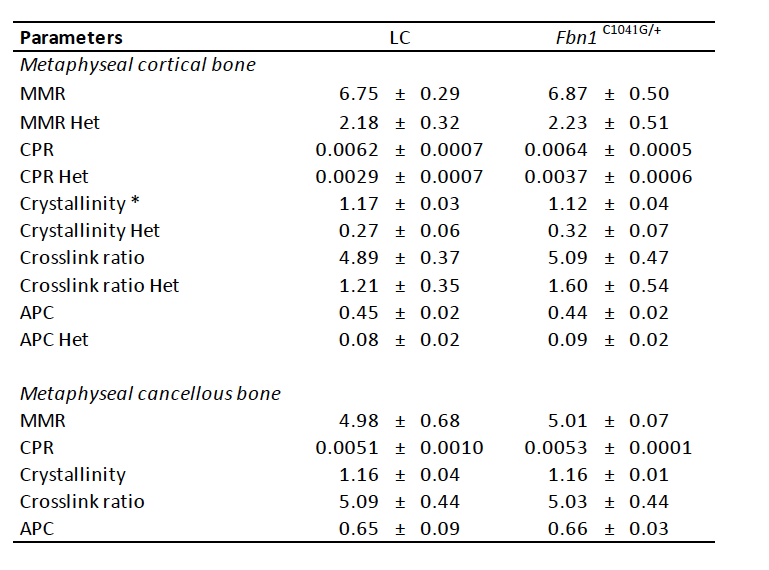


**Supplemental Table 4:** **Lacunar parameters in metaphyseal and diaphyseal cortical bone**. Synchrotron µCT was used to investigate characteristics of osteocyte lacunae in 26-week-old female littermate (LC) and *Fbn1^C1041G/+^* mice. Data are presented as mean ± standard deviation. ANOVA main effects: ^a^genotype (*Fbn1^C1041G/+^*, LC mice), ^b^region (endocortical, periosteal, intracortical) and interactions:  ^c^region+genotype. *Comparison between genotype was performed using a Tukey-Kramer post-hoc test. *An independent T-test was used to investigate differences in the full volume. Significance for all tests was set at p ≤ 0.05.


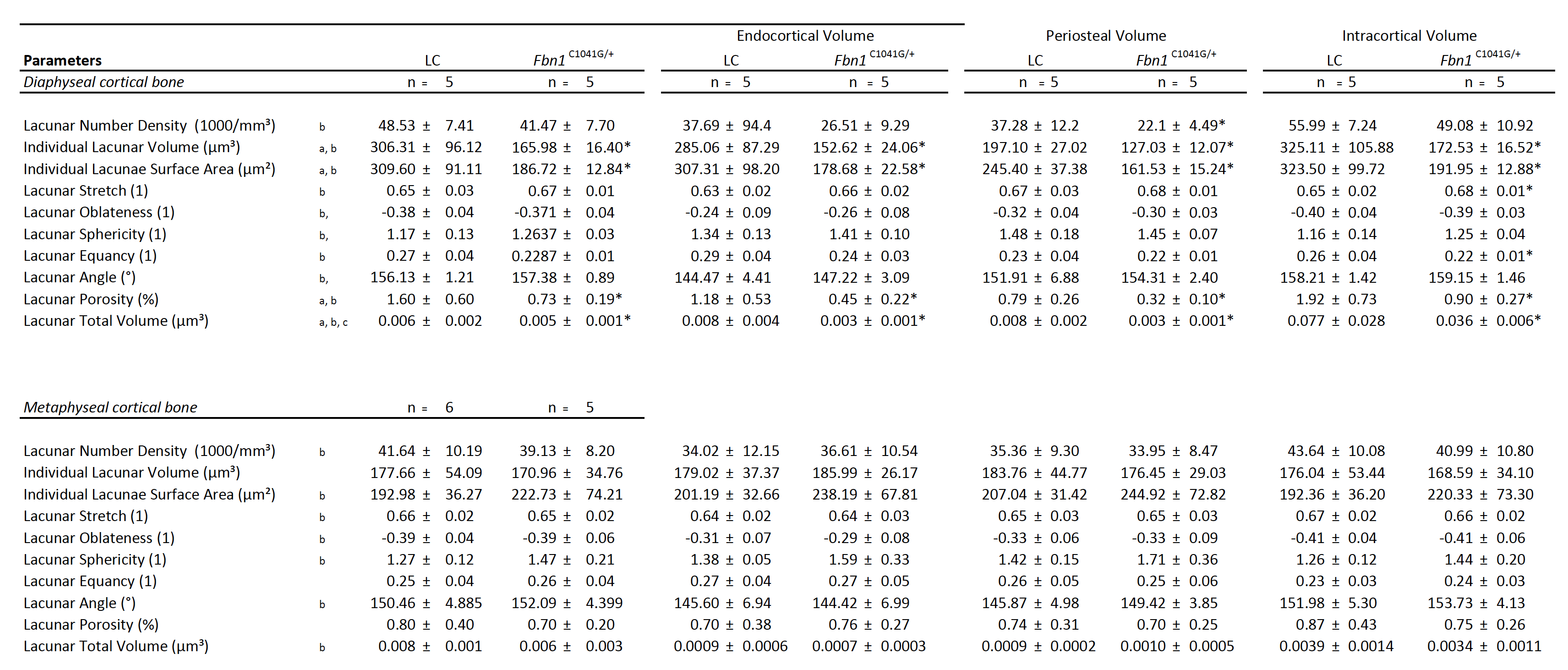


**Supplemental Table 5:** **Vascular parameters in metaphyseal and diaphyseal cortical bone.** Synchrotron µCT was used to investigate characteristics of vasculature in 26-week-old female littermate control (LC) and *Fbn1^C1041G/+^* mice. Data are presented as mean ± standard deviation. ANOVA main effects: ^a^genotype (*Fbn1^C1041G/+^*, LC mice), ^b^region (endocortical, periosteal, intracortical) and interactions:  ^c^region+genotype. *Comparison between genotype was performed using a Tukey-Kramer post-hoc test. *An independent T-test was used to investigate differences in the full volume. Significance for all tests was set at p ≤ 0.05.


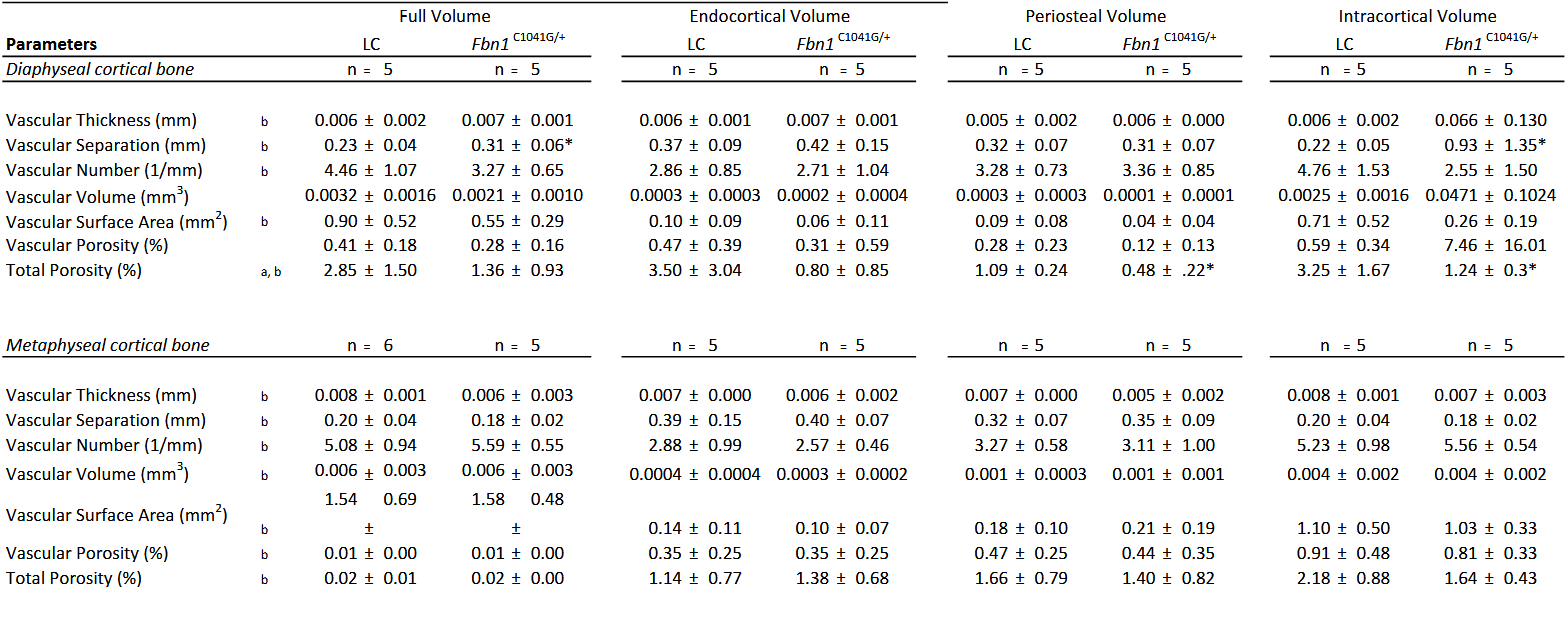

Supplement: Supplementary Data 1 [file mmc1.docx]
